# Dual-Wavelength Laser Speckle Contrast Imaging (dwLSCI) Improves Chronic Measurement of Superficial Blood Flow in Hands

**DOI:** 10.3390/s17122811

**Published:** 2017-12-05

**Authors:** Lingke Zhang, Li Ding, Miao Li, Xiaoli Zhang, Diansan Su, Jie Jia, Peng Miao

**Affiliations:** 1 Biomedical Engineering Institute, School of Communication and Information Engineering, Shanghai University, Shanghai 200444, China; lkzhang@i.shu.edu.cn (L.Z.); Azura@i.shu.edu.cn (M.L.); pengmiao@shu.edu.cn (P.M.); 2 Department of Rehabilitation Medicine, Huashan Hospital, Fudan University, Shanghai 200040, China; 16111220060@fudan.edu.cn (L.D.); xiaolizhang01@126.com (X.Z.); shannonjj@126.com (J.J.); 3 Department of Anesthesiology, Renji Hospital, School of Medicine, Shanghai Jiao Tong University, Shanghai 200127, China

**Keywords:** laser speckle contrast imaging, hand perfusion sensing, stroke rehabilitation, dual-wavelength

## Abstract

Laser speckle contrast imaging (LSCI) has been widely used to determine blood flow and perfusion in biological tissues. The physical model of traditional LSCI ignores the effects of scattering property distribution in relation to speckle correlation time *τ_c_* and blood flow *v*, which further results in biased estimation. In this study, we developed a dual-wavelength laser speckle contrast imaging (dwLSCI) method and a portable device for imaging the blood flow and tissue perfusion in human hands. Experimental data showed that dwLSCI could retrieve the vein vasculatures under the surface skin, and it further provided accurate measurements of vein blood flow signals, tissue perfusion signals, and fingertip perfusion signals, which assist with assessments of rehabilitation therapy for stroke patients. Fingertip perfusion signals demonstrated better performance in early assessments, while vein blood flow signals assisted the Fugl–Meyer Assessment Scale (FMA) and the Wolf Motor Function Test (WMFT) behavior assessments. As a general noninvasive imaging method, dwLSCI can be applied in clinical studies related to hand functions combined with behavior assessments.

## 1. Introduction

Changes of hand superficial vascular perfusion are direct indicators of diseases, such as vasculitis, Raynaud’s syndrome, and hemangioma [1]. Perfusion in hand surfaces is related to the vasculature network, blood flow, blood pressure, and tissue metabolism [2]. These physiological parameters are also useful in hand function rehabilitations. For example, stroke patients suffering from brain function impairment may have hand and finger motion dysfunction [3,4,5]. Muscle atrophy and venous return stagnation in hands are two major reasons for abnormal perfusion [6]. In clinical practice, different assessments, such as the Fugl–Meyer Assessment Scale (FMA) [7] and the Wolf Motor Function Test (WMFT) [8], are widely used to estimate rehabilitation effects by scoring the completion of specific training tasks. These tests are performed under the direction of clinicians, and patients with better rehabilitation effects obtain higher scores. Such tests provide qualitative assessments but have sensitivity and robustness limitations. Monitoring hand blood flow and perfusion provides more physiological information and further benefits clinical diagnoses and rehabilitation planning.

Currently, the most widely used methods for the measurement of hand perfusion are magnetic resonance angiography (MRA) [1,9,10,11], laser Doppler flowmetry (LDF) [12,13,14], and laser speckle contrast imaging (LSCI) [15,16,17,18,19]. The MRA method has the ability to visualize small caliber target vessels [1]. However, it requires a contrast agent and only works for the arterial system [1,10]. The LDF method provides a contrast-agent-free and more reliable measurement of skin tissue perfusion but is limited in both spatial and temporal resolution [12,15]. LSCI provides a non-invasive perfusion assessment with higher spatial and temporal resolution in a full-field manner [15,18,19]. Furthermore, the LSCI system is cost-effective and outputs real-time monitoring [15]. However, the current LSCI reconstruction cannot reveal accurate hand surface perfusion due to the uneven distribution of scattering properties, which further leads to difficulties in explaining hand perfusion changes.

In recent years, the methods of multi-wavelength LSCI have been proposed. Jeong has developed a simplified multi-wavelength LSCI system, which can guide laser diodes of 450 nm and 780 nm to generate speckle patterns with a single holographic optical element [20]. Dunn combined laser speckle contrast imaging and multi-wavelength imaging to monitor the changes of total hemoglobin concentration, blood flow, and metabolic oxygen [21,22]. Multi-wavelength LSCI has also been developed to measure multiple hemodynamic responses [23,24]. However, most of the techniques have been used for cerebral blood flow (CBF) and metabolism measurements.

In this study, we propose a new strategy for retrieving a corrected blood flow distribution by decoupling the spatial distribution of the scattering coefficient. This dual-wavelength laser speckle imaging (dwLSCI) provides better visualization of the vein system and accurate measurement of superficial perfusion in hands. We also used this method to evaluate hand rehabilitation in stroke patients.

## 2. Methods and Experiments

### 2.1. Theory

Traditional LSCI utilizes speckle contrast K=σ/μ to estimate the speed v of scattering particles, e.g., red blood cells in blood flow. Usually, sequentially recorded speckle images under coherent light illumination are analyzed, and contrast images $K^{2}\left(x,\;y\right)$ are reconstructed to represent the blood flow distribution map (Equation (1)).

(1) $$K^{2}=\beta \left\{\tau_{c}/T+{\left(\tau_{c}/T\right)}^{2}\left[exp\left(-2T/\tau_{c}\right)-1\right]/2\right\}$$

where $\tau_{c}$ is the correlation time, which is assumed to be inversely proportional to the speed of the scattering particles v. β represents for the loss of correlation related to the ratio of the detector size to the speckle size and polarization, and T is the exposure time.

In practice, as a measure of flux [25], the parameter $\tau_{c}$ provides useful physiological information in clinical applications. Recent studies in the theoretic framework of LSCI show that local scattering coefficients of moving scatters, i.e., $\mu_{s}$, scale the proportional relation between $\tau_{c}$ and v [26,27]:

(2) $$1/\tau_{c}\propto \mu_{s}\cdot v.$$

To obtain the correct estimation of the blood flow distribution map v(x,y), the scattering coefficients map $\mu_{s}\left(x,y\right)$ should be measured simultaneously. Based on a study by Twersky, $\mu_{s}\left(x,y\right)$ is determined by the local volume fraction H(x,y) of RBC in each pixel (Equation (3)).

(3) $$\mu_{s}\left(x,y\right)=\frac{\sigma_{s}}{V}H\left(x,y\right)\left(1-H\left(x,y\right)\right)$$

where $\sigma_{s}$ and V are the scattering cross section and volume of single RBC, respectively.

In the imaging of the biological tissue, RBCs carry two different kinds of hemoglobin (oxy- and deoxy-hemoglobins). Thus, H(x,y) can be estimated from the total hemoglobin concentration $C_{HbT}\left(x,y\right)$ (Equation (4)).

(4) $$H\left(x,y\right)\propto C_{HbT}\left(x,y\right).$$

Various methods have been developed to obtain hemoglobin distribution by spectral imaging techniques and are simultaneously used in LSCI applications [21,28,29,30]. In this study, we developed a strategy to retrieve the blood flow information based on the isosbestic point (equal absorption wavelength) located at 805 nm (near-infrared range). Although there are isosbestic points located in visible light, near-infrared light provides better penetration in human hand applications.

Under 805 nm light illumination, the reflectance intensity map $I_{805nm}\left(x,y\right)$ of the imaging area is determined by the absorption of the total hemoglobin based on the Beer–Lambert law (Equation (5))

(5) $$I_{805nm}\left(x,y\right)=I_{0}\left(x,y\right)e^{-\mu_{a}\left(x,y\right)\cdot L}$$

where $I_{0}\left(x,y\right)$ is the intensity map of incident light, $\mu_{a}$ is the absorption coefficient and is proportional to $C_{HbT}\left(x,y\right)$, and L is the averaged light path-length in tissue.

Combining Equations (3)–(5), the estimation of superficial blood flow v(x,y) in a hand is conducted in Equation (6).

(6) $$\frac{1}{v\left(x,y\right)}\propto \frac{\tau_{c}\left(x,y\right)\sigma_{s}\left[lnI_{0}\left(x,y\right)-lnI_{805nm}\left(x,y\right)\right]}{Vc^{2}L^{2}}\left\{cL-\left[lnI_{0}\left(x,y\right)-lnI_{805nm}\left(x,y\right)\right]\right\}$$

where c is a constant coefficient for the normalization purpose.

### 2.2. Equipment and Data Processing

**Figure 1:**
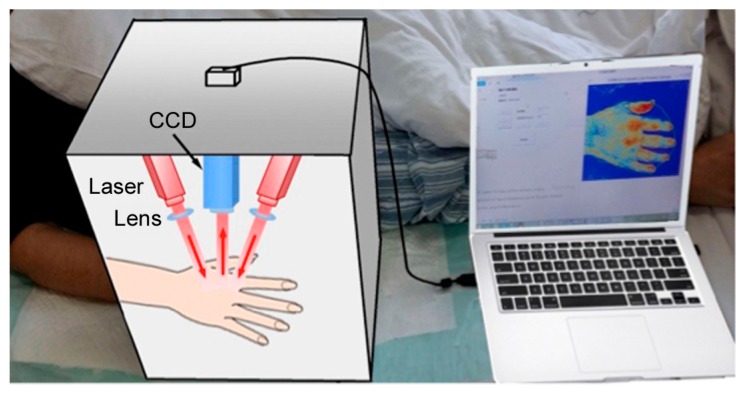
Portable imaging box developed for the imaging data acquisition.

In this study, we designed a portable imaging box to perform the imaging data acquisition (Figure 1). The system uses divergent laser beams with wavelengths of 785 nm and 805 nm to illuminate the skin. The distance between the camera and the hand was kept at 21 cm, and the scanning area was set to correspond to a 23 cm × 17 cm area.

A board-level programmable TTL signal generator was used to synchronize the exposure of the CCD camera (scA1300-32fm, Basler Inc., Ahrensburg, Germany) and repeated illuminations of laser light 785 nm (90 mW, L785P090, Thorlabs Inc., Newton, NJ, USA) and 805 nm (500 mW, ML620G40, Thorlabs Inc., Newton, NJ, USA). The CCD camera recorded 12-bit images with a resolution of 1296 × 966, an exposure time of 20 ms, and a frame rate of 30 fps. The imaging parameters of exposure time and frame rate work well for the baseline perfusion imaging of human hands at different periods during the rehabilitation program. In fact, a shorter exposure time and higher frame rate can further improve imaging sensitivity, but greater laser light power, which may introduce heating effects, is needed. Furthermore, a camera with a high frame rate is usually expensive. High sensitivity is needed when imaging transient changes in blood flow or perfusion, e.g., in the functional imaging of the CBF.

After recording speckle images under 780 nm illuminations, contrast images K(x,y) were obtained using LASCA theory [31]. Both spatial [31] and temporal [32] estimation methods can be applied in the contrast calculation. The spatial LASCA method improves temporal resolution under the cost of spatial resolution, while the temporal LASCA method loses temporal resolution in order to obtain a sufficient signal/noise ratio (SNR) (usually more than 25 speckle frames are required to calculate one contrast image). In this study, the spatio-temporal estimation method [33] was used in the calculation of contrast images (every five speckle images output one contrast image), with which, by using a random process estimator to suppress the noise in these images, a sufficient SNR could be obtained and the temporal resolution maintained. After that, $\tau_{c}\left(x,y\right)$ was calculated according to Equation (1), here, we chose β=0.5 [34].

**Figure 2:**
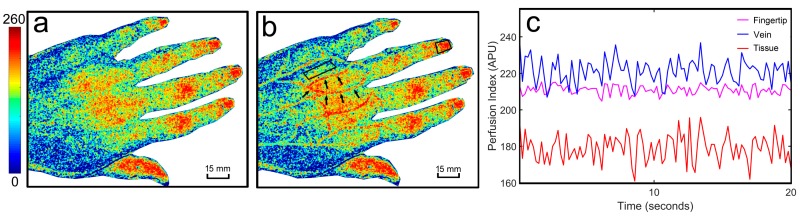
Example of superficial hand blood flow in one stroke patient. (**a**) A traditional LSCI image, which cannot reveal the vein vasculature details; (**b**) visible vascular information using dwLSCI and the ROIs we selected in the vein, its surrounding tissue and its supplying fingertip; (**c**) the perfusion index signals in the selected ROIs.

For the recorded images under 805 nm illuminations, a temporal average operation was performed as the first step for every five images, outputting one $I_{805nm}\left(x,y\right)$. Then, the right side of Equation (6) was calculated, and the perfusion index image (Figure 2b) was obtained. Here, the perfusion index was proportional to the blood flow distribution map v(x,y), which is the reciprocal of the calculated value of the right side in Equation (6).

In the calculation of Equation (6), L is the averaged light path-length in the tissue. For the human hand, the 805 nm laser light (500 mW) can provide a penetration depth of about 2~3 mm [35], so we applied *L* = 2.5 mm to the calculation. Moreover, typical human red blood cells have an average volume of about V=90 fL [36], with a scattering cross section of about $\sigma_{s}=63.82\;{\mathrm{um}}^{2}$ [37]. The intensity image $I_{0}\left(x,y\right)$ for the 805 nm light source was measured using white paper as the non-absorbing object.

### 2.3. Experiments

The Ethics Review Board of the Huashan Hospital approved this study (2013163). All patients enrolled in this study suffered from lacunar infarction without sequelae (confirmed by CT or MRI). All the diagnoses are consistent with the main points of a cerebrovascular disease diagnosis adopted by Fourth National Cerebrovascular Disease Conference 1995. The exclusion criteria were as follows: (i) peripheral neuropathy of upper limbs, (ii) severe cardiopulmonary diseases, (iii) MMSE scale results revealing dementia, (iv) presence of movement disorders, (v) history of cognitive disorders, neuropsychiatric disorders, or drug abuse, (vi) unacceptable use of electro-acupuncture treatment (e.g., injured skin or syncope), and (vii) deterioration of the disease (e.g., the emergence of new infarction or massive cerebral infarction).

Thirty eligible stoke patients were enrolled between the ages of 40 and 60 who suffered first-episode hand dysfunction. According to the degree of hand functional injury, the 30 patients were divided into three grades: Brunnstrom I~II, Brunnstrom III~IV, and Brunnstrom V. Patients of different grades took different rehabilitation therapy. The patients of Brunnstrom I~II whose hand dysfunction was the lightest could be trained and gradually closed to the normal active movement. Seven patients in the grade of Brunnstrom I~II took the same rehabilitation program, and they were imaged before, during, and after the six weeks of therapy (on Day 2, Week 1, Week 2, Week 4, Week 6, and Week 12). To assess the rehabilitation of hand function of stroke patients, the Fugl–Meyer Assessment Scale (FMA) and the Wolf Motor Function Test (WMFT) were applied together with the imaging experiment. The rehabilitation program included routine rehabilitation and additional electro-acupuncture for six weeks. A G6805 electro-acupuncture device (10 Hz, 20 min one session) and disposable acupuncture needles (size No. 28, length 1.5 inches) were used to perform electro-acupuncture stimulation.

## 3. Results

### 3.1. The dwLSCI Improvement of Superficial Blood Flow Imaging

The vein vasculature details cannot be revealed by using traditional LSCI in Figure 2a. dwLSCI can increase imaging sensitivity after correcting effects from the non-uniform distribution of local scattering properties (Figure 2b). However, the dwLSCI method significantly improves the superficial vascular imaging and overcomes the limitations of traditional LSCI (Figure 2b). As a 2D imaging method, different ROIs can be outlined to quantitatively analyze blood flow and tissue perfusion (Figure 2b).

To investigate the changes of blood flow and tissue perfusion during stroke rehabilitation, we selected the ROIs covering a vein, its surrounding tissue, and its supplying fingertips (see the ring finger in Figure 2b as an example). Figure 2c illustrates the perfusion index signals in the selected ROIs. The signal in the vein area shows the highest perfusion level during the imaging session. The signal in the fingertip area, compared with that in the vein, has a slightly smaller baseline and less variation. Furthermore, the tissue perfusion signal keeps the lowest baseline and shows the largest fluctuations.

It should be noted that a semi-transparent nail covers each fingertip, while skin covers the other ROIs. These physiological differences bias the baseline in the perfusion signals. For tissue perfusion estimation, a semi-transparent nail provides better accuracy and higher sensitivity in the current imaging configuration.

### 3.2. Healthy Volunteers Demonstrate Higher Superficial Perfusion of Hands than Stroke Patients

**Figure 3:**
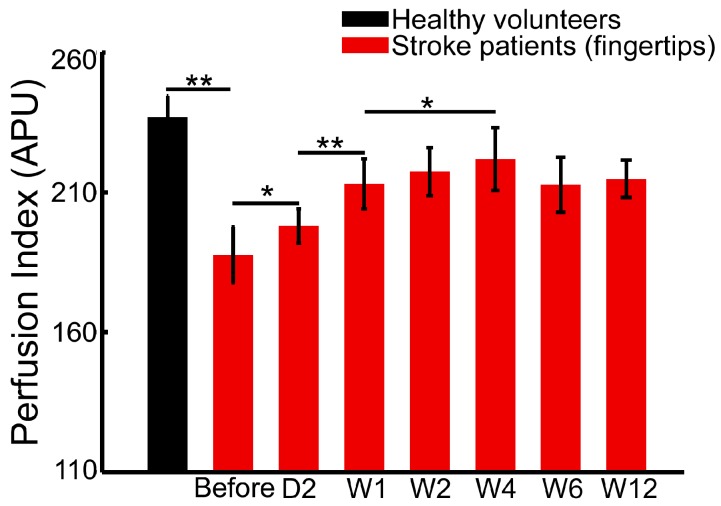
Averaged perfusion changes in the fingertips of healthy volunteers and stroke patients before, during, and after six weeks of rehabilitation therapy (* *p* < 0.05; ** *p* < 0.01).

**Figure 4:**
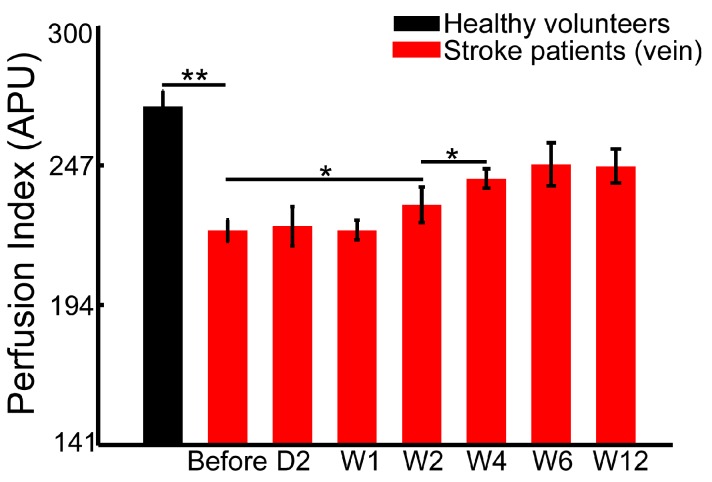
Statistical analysis of the superficial blood flow signal in hand veins of healthy volunteers and stroke patients (* *p* < 0.05; ** *p* < 0.01).

**Figure 5:**
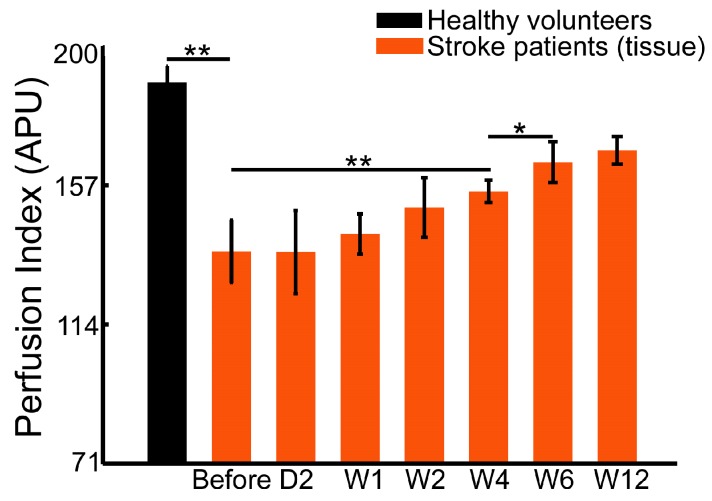
Tissue perfusion signals in healthy volunteers and patients at different stages of rehabilitation (* *p* < 0.05; ** *p* < 0.01).

In this study, we monitored the blood flow and tissue perfusion in seven healthy volunteers and seven stroke patients. The imaging experiments for stroke patients were performed continuously over six weeks of rehabilitation therapy. Figure 3, Figure 4 and Figure 5 shows the averaged perfusion index in different groups, areas and rehabilitation stages. The signals of healthy volunteers range from 196 to 235 APU in fingertips, from 170 to 193 APU in tissue, and from 227 to 255 APU in veins. For stroke patients, the range of the perfusion index is obviously smaller, and is from 110 to 228 APU in nails, from 71 to 158 APU in tissue, and from 141 to 226 APU in veins.

The perfusion signal of fingertips in healthy volunteers (219.9 ± 13.1 APU) is significantly higher than that of the patients before (134.6 ± 17.2 APU, *p* < 0.01) and after the 6-week rehabilitation therapy (at 12 weeks, 181.6 ± 11.5 APU, *p* < 0.01). Similarly, the blood flow signal in the superficial veins in healthy volunteer hands (242.3 ± 11.1 APU) is significantly higher than that of patients before (153.1 ± 7.8 APU, *p* < 0.01) and after therapy (at 12 weeks, 199.4 ± 12.1 APU, *p* < 0.01). The tissue perfusion signal in healthy volunteers (182.4 ± 7.9 APU) is also significantly higher than that in patients (101.6 ± 15.0 APU before therapy, and 150.0 ± 6.6 APU at 12 weeks, *p* < 0.01).

### 3.3. Early Stage Rehabilitation Therapy Improves Fingertip Perfusion

Figure 3 shows the averaged perfusion changes in the fingertips of stroke patients before and after the 6-week rehabilitation therapy. The perfusion level in fingertips shows an early response to therapy (on Day 2, 152.3 ± 10.7 APU) compared with the status before therapy (134.6 ± 17.2 APU, *p* < 0.05). Moreover, the perfusion level continuously increased after one week of therapy (178.4 ± 15.5 APU) compared with the perfusion level on Day 2 (*p* < 0.01). Later, a significantly higher perfusion index was measured at Week 4 of therapy (193.9 ± 19.5 APU, *p* < 0.05) compared with Week 1. There were no significant increases or changes after four weeks of therapy.

For stroke patients with hand dysfunction, long-term inactivity of the hand muscles leads to insufficient blood supply and poor microcirculation. Therefore, compared with the healthy volunteers, there was significant lower tissue perfusion in the fingertips. However, the changes of tissue perfusion in fingertips show that inadequate perfusion can be notably improved after rehabilitation therapy.

### 3.4. Blood Flow in the Superficial Veins and Surrounding Tissue Perfusion in Hands Demonstrates Different Recovering Patterns during Rehabilitation Therapy

Unlike fingertips, the blood flow signal in the superficial veins in hands shows no obvious changes within a week of therapy (Figure 4). The blood flow after two weeks of rehabilitation, compared with that before therapy (153.1 ± 7.8 APU, *p* < 0.05), was significantly improved (171.7 ± 12.7 APU). The vein blood flow signal after four weeks of rehabilitation (190.6 ± 7.0 APU) shows more improvement (*p* < 0.05). Consistent with the tissue perfusion signals in fingertips, the blood flow signal in the superficial vein in hands remains stable after four weeks of rehabilitation therapy.

The mean perfusion index in tissue is shown in Figure 5. Similar to the vein area, there was no significant difference during the first four weeks of therapy. The tissue perfusion at four weeks, when compared with that before therapy (101.6 ± 15.0 APU, *p* < 0.01), is greatly improved (130.3 ± 5.5 APU). In addition, perfusion after six weeks of therapy (144.3 ± 9.8 APU) is even higher than that after four weeks of therapy (*p* < 0.05). The extent of tissue perfusion improvement is different from that in veins and fingertips.

## 4. Discussion

### 4.1. FMA and WMFT Demonstrate Limitations in the Early Assessment of Rehabilitation Therapy

In clinical practice, the Fugl–Meyer Assessment Scale (FMA) and the Wolf Motor Function Test (WMFT) are typically applied to assess the hand and upper limb functions in the rehabilitation of stroke patients. FMA and WMFT provide quantitative assessments of motor behaviors but with limitations, including insufficient sensitivity in early therapy and less robustness in testing procedures. The dwLSCI method developed in this study can help to improve the FMA and WMFT assessments by providing tissue perfusion information.

**Figure 6:**
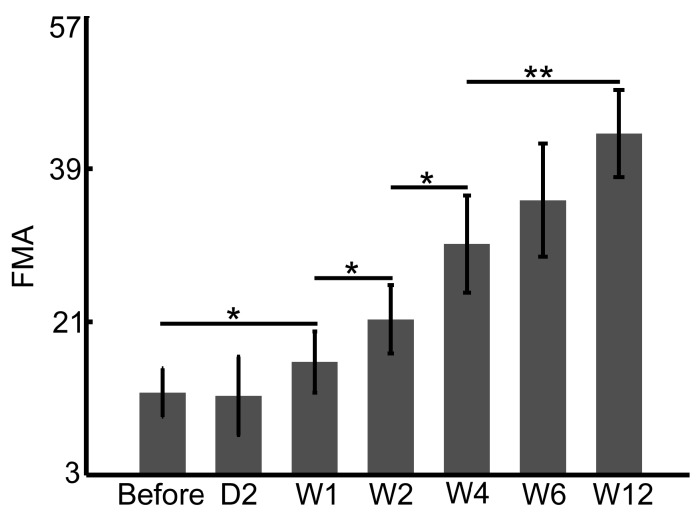
Fugl–Meyer Assessment Scale (FMA) monitors the rehabilitation procedures in stroke patients (* *p* < 0.05; ** *p* < 0.01).

**Figure 7:**
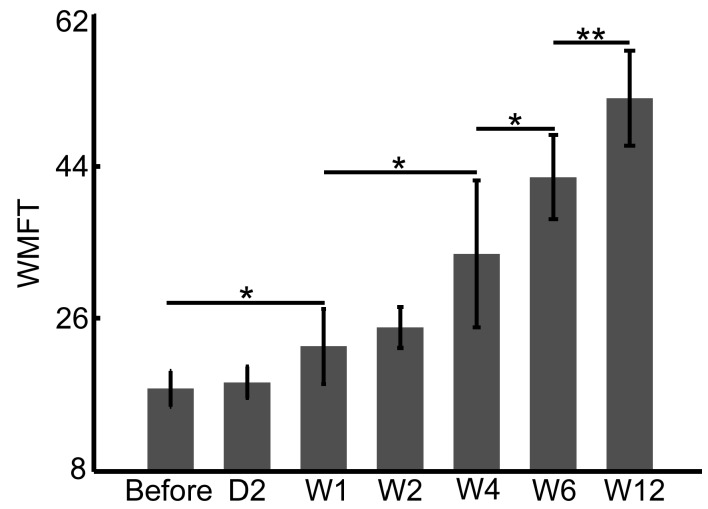
Statistical analysis of the Wolf Motor Function Test (WMFT) scores in stroke patients during six weeks of therapy (* *p* < 0.05; ** *p* < 0.01).

Figure 6 and Figure 7 show the FMA and WMFT results, respectively, of stroke patients in this study before and after six weeks of therapy. The significant difference appears after one week therapy, both in FMA (10.7 ± 3.1 APU before vs. 14.7 ± 4.0 APU Week 1, *p* < 0.05, Figure 6) and WMFT (11.1 ± 2.4 APU before vs. 16.9 ± 5.1 APU Week 1, *p* < 0.05, Figure 7). Both scores of FMA and WMFT rise gradually over the six weeks of therapy; however, there are subtle differences in the statistical results between FMA and WMFT. FMA evaluates changes in sports patterns, and the WMFT is based on the changes in upper limb function. However, both assessments can monitor the rehabilitation process.

### 4.2. Perfusion Signal in Fingertips Provides More Sensitive Monitoring of Early Rehabilitation

Compared to the skin, the semi-transparent nail provides better imaging conditions in tissue perfusion assessment during rehabilitation therapy. The detected baseline of perfusion signals in fingertips is considerably higher than it is in tissue (Figure 3 vs. Figure 5). Therefore, only this signal shows early responses to therapy (there is a statistical difference between before therapy and after two days of therapy in Figure 3). The fingertip is rich in capillaries, which act as the microvasculature network supplied by arteries and veins, and it is more sensitive to changes in blood flow and perfusion. This sensitivity makes the fingertip signal an effective indicator for early assessment.

The perfusion signal of the tissue area, compared with those of the other two areas, is the lowest, and the signals of the veins and the surrounding tissue do not show significant changes in the early therapy period. The signals of the fingertips (Figure 3) and veins (Figure 4) remain stable after four weeks of rehabilitation therapy, whereas the perfusion signal of the tissue does not (Figure 5). Additionally, the perfusion signals in the tissue areas continually increase as rehabilitation therapy progresses. However, the increasing degree of perfusion is much lower than those of the fingertips and veins because of the lowest baseline. Therefore, the perfusion signal of the tissue area cannot be regarded as an effective and sensitive indicator.

Although the skin affects imaging performance, the blood flow signal in veins still plays an important role in the detection of the superficial blood flow in hands. There are obvious changes in vein blood flow signals between Week 2 and Week 4, and it tends to stabilize after Week 4. These patterns are correlated with the motor function improvement assessed by FMA and WMFT.

### 4.3. Perfusion and Flow Measurements Assist the Quantitative Assessment of Behavior Testing

Combining quantitative behavior tests (FMA and/or WMFT) with the perfusion signals in veins and the flow signals in fingertips helps to monitor rehabilitation therapy. As a sensitivity indicator, fingertip perfusion signals have a diagnostic ability for monitoring early rehabilitation, which cannot be assessed by the behavior test. This parameter is helpful for the early assessment and dynamic adjustment of rehabilitation programs. The vein flow signal combined with behavioral tests can provide more robust assessments in individual therapy cases. 

As a general imaging method, dwLSCI can also be applied in studies of the pathogenesis of circulatory system diseases. The perfusion and blood flow signals will form new indicators and further assist in the diagnosis of diseases, such as vasculitis, Raynaud’s syndrome, disseminated Candida infection, and hemangioma.

## 5. Conclusions

Chronic measurement of superficial blood flow and tissue perfusion in hands is of great importance for monitoring their physiological and pathological status. In this study, the dwLSCI method is proposed to image and measure the hand’s superficial blood flow and tissue perfusion. We applied the new method to the assessments of hand functional rehabilitation in stroke patients. Our study suggested the fingertip perfusion signal as the sensitive indicator for early rehabilitation assessments. Furthermore, dwLSCI combined with traditional behavior tests will benefit the overall evaluations of clinical rehabilitation.

## References

[1] Huerta T.E., Norton P.T., Chhabra B., Drake D.B., Arnold P.B., Housseini A.M., Hagspiel K.D. (2009). Comprehensive Magnetic resonance imaging of the hand and forearm vasculature at 3 Tesla using time-resolved angiography with stochastic trajectories (TWIST): Preliminary clinical results. Proc. Int. Soc. Magn. Reson. Med..

[2] Sosa J.M., Nielsen N.D., Vignes S.M., Chen T.G., Shevkoplyas S.S. (2014). The relationship between red blood cell deformability metrics and perfusion of an artificial microvascular network. Clin. Hemorheol. Microcirc..

[3] Budimkic M.S., Pekmezovic T., Beslac-Bumbasirevic L., Ercegovac M., Berisavac I., Stanarcevic P., Padjen V., Jovanovic D.R. (2015). Long-term medication persistence in stroke patients treated with intravenous thrombolysis. Clin. Neurol. Neurosurg..

[4] Saeki S., Chisaka H., Hachisuka K. (2005). Life satisfaction and functional disabilities in long-term survivors after first stroke. J. UOEH.

[5] McKevitt C., Fudge N., Redfern J., Sheldenkar A., Crichton S., Rudd A.R., Forster A., Young J., Nazareth I., Silver L.E. (2011). Self-reported long-term needs after stroke. Stroke.

[6] Nobutoki T., Ihara T. (2015). Early disruption of neurovascular units and microcirculatory dysfunction in the spinal cord in spinal muscular atrophy type I. Med. Hypotheses.

[7] Van der Lee J.H., Beckerman H., Lankhorst G.J., Bouter L.M. (2001). The responsiveness of the Action Research Arm test and the Fugl-Meyer Assessment scale in chronic stroke patients. J. Rehabil. Med..

[8] Padovani C., Pires C.V.G., Ferreira F.P.C., Borin G., Filippo T.R.M., Imamura M., Rosa C.D.P., Battistella L.R. (2016). Application of the Fugl-Meyer Assessment (FMA) and the Wolf Motor Function Test (WMFT) in the recovery of upper limb function in patients after chronic stroke: A literature review. Acta Fisiatr..

[9] Gutzeit A., Eckhardt B., Beranek J., Wentz K.U., Willemse E., Jenelten R., Binkert C.A., Froehlich J.M. (2010). Clinical experience in timed arterial compression contrast-enhanced magnetic resonance angiography of the hand. Can. Assoc. Radiol. J..

[10] Fan Z., Hodnett P.A., Davarpanah A.H., Scanlon T.G., Sheehan J.J., Varga J., Carr J.C., Li D. (2011). Noncontrast MR angiography of the hand: Improved arterial conspicuity by multi-directional flow-sensitive dephasing magnetization preparation in 3D Balanced SSFP Imaging. Investig. Radiol..

[11] Connel D.A., Koulouris G., Thorn D.A., Potter H.G. (2002). Contrast-enhanced MR angiography of the hand. RadioGraphics.

[12] Gardner-Medwin J.M., Taylor J.Y., Macdonald I.A., Powell R.J. (1997). An investigation into variability in microvascular skin blood flow and the responses to transdermal delivery of acetylcholine at different sites in the forearm and hand. Br. J. Clin. Pharmacol..

[13] He D., Nguyen H.C., Hayes-Gill B.R., Zhu Y., Crowe J.A., Gill C., Clough G.F., Morgan S.P. (2013). Laser Doppler blood flow imaging using a CMOS imaging sensor with on-chip signal processing. Sensors.

[14] Iwasaki W., Nogami H., Takeuchi S., Furue M., Higurashi E., Sawada R. (2015). Detection of site-specific blood flow variation in humans during running by a wearable laser Doppler flowmeter. Sensors.

[15] Pauling J.D., Shipley J.A., Raper S., Watson M.L., Ward S.G., Harris N.D., McHugh N.J. (2012). Comparison of infrared thermography and laser speckle contrast imaging for the dynamic assessment of digital microvascular function. Microvasc. Res..

[16] Lee J., Moon S., Lim J., Gwak M.-J., Kim J.G., Chung E., Lee J.-H. (2017). Imaging of the finger vein and blood flow for anti-spoofing authentication using a laser and a MEMS scanner. Sensors.

[17] Zötterman J., Bergkvist M., Iredahl F., Tesselaar E., Farnebo S. (2016). Monitoring of partial and full venous outflow obstruction in a porcine flap model using laser speckle contrast imaging. J. Plast. Reconstr. Aesthet. Surg..

[18] Pauling J.D., Shipley J.A., Hart D.J., McGrogan A., McHugh N.J. (2015). Use of laser speckle contrast imaging to assess digital microvascular function in primary Raynaud phenomenon and systemic sclerosis: A comparison using the Raynaud condition score diary. J. Rheumatol..

[19] Iredahl F., Löfberg A., Sjöberg F., Farnebo S., Tesselaar E. (2015). Non-invasive measurement of skin microvascular response during pharmacological and physiological provocations. PLoS ONE.

[20] Jeong Y., Li G., Lee D., Lee B. (2016). Simplified multi-wavelength laser speckle contrast imaging system by using single holographic optical element. Imaging Appl. Opt..

[21] Dunn A.K., Devor A., Bolay H., Andermann M.L., Moskowitz M.A., Dale A.M., Boas D.A. (2003). Simultaneous imaging of total cerebral hemoglobin concentration oxygenation and blood flow during functional activation. Opt. Lett..

[22] Dunn A.K., Devor A., Dale A.M., Boas D.A. High resolution imaging of the hemodynamic and metabolic response to functional activation. Proceedings of the Biomedical Topical Meeting.

[23] Chen W., Park K., Pan Y., Du C. (2015). Abnormal hemodynamic response to forepaw stimulation in rat brain after cocaine injection. Optical Techniques in Neurosurgery, Neurophotonics, and Optogenetics II.

[24] Chen W., Park K., Volkow N., Pan Y., Du C. (2016). Cocaine-induced abnormal cerebral hemodynamic responses to forepaw stimulation assessed by integrated multi-wavelength spectroimaging and laser speckle contrast imaging. IEEE J. Sel. Top. Quantum Electron..

[25] Khaksari K., Kirkpatrick S.J. (2016). Laser speckle contrast imaging is sensitive to advective flux. J. Biomed. Opt..

[26] Miao P., Chao Z., Feng S., Yu H., Ji Y., Li N., Thakor N.V. (2015). Local scattering property scales flow speed estimation in laser speckle contrast imaging. Laser Phys. Lett..

[27] Khaksari K., Kirkpatrick S.J. (2016). Combined effects of scattering and absorption on laser speckle contrast imaging. J. Biomed. Opt..

[28] Luo Z., Yuan Z., Pan Y., Du C. (2009). Simultaneous imaging of cortical hemodynamics and blood oxygenation change during cerebral ischemia using dual-wavelength laser speckle contrast imaging. Opt. Lett..

[29] Qin J., Shi L., Dziennis S., Reif R., Wang R.K. (2012). Fast synchronized dual-wavelength laser speckle imaging system for monitoring hemodynamic changes in a stroke mouse model. Opt. Lett..

[30] Wang J., Wang Y., Li B., Feng D., Lu J., Luo Q., Li P. (2013). Dual-wavelength laser speckle imaging to simultaneously access blood flow, blood volume, and oxygenation using a color CCD camera. Opt. Lett..

[31] Briers J.D., Webster S. (1996). Laser speckle contrast analysis (LASCA): A nonscanning, full-field technique for monitoring capillary blood flow. J. Biomed. Opt..

[32] Li P., Ni S., Zhang L., Zeng S., Luo Q. (2006). Imaging cerebral blood flow through the intact rat skull with temporal laser speckle imaging. Opt. Lett..

[33] Miao P., Li N., Thakor N.V., Tong S. (2010). Random process estimator for laser speckle imaging of cerebral blood flow. Opt. Express.

[34] Boas D.A., Dunn A.K. (2010). Laser speckle contrast imaging in biomedical optics. J. Biomed. Opt..

[35] Avci P., Gupta A., Sadasivam M., Vecchio D., Pam Z., Pam N., Hamblin M.R. (2013). Low-level laser (light) therapy (LLLT) in skin: Stimulating, healing, restoring. Semin. Cutan. Med. Surg..

[36] McLaren C.E., Brittenham G.M., Hasselblad V. (1987). Statistical and graphical evaluation of erythrocyte volume distributions. Am. J. Physiol..

[37] Steinke J.M., Shepherd A.P. (1988). Comparison of Mie theory and the light scattering of red blood cells. Appl. Opt..

